# 

**Published:** 1888-04

**Authors:** 


### THE MEDICAL ASSOCIATION OF GEORGIA.

The thirty-ninth annual meeting of the Medical Association of Georgia will be held in Rome, April 18th, 19th and 20th. Measures of the utmost importance will be considered, and it is therefore desirable that every one who can should be present. If YOU are not already a member, the desirability of becoming one is urged upon you. This old and honored institution has not heretofore secured the co-operation which it deserves. A little effort on the part of its friends will largely increase the roll of its membership.

The railroad rates to members, and those becoming members, have been fixed at one and one-third fares for the round trip. Pay full fare going, getting from the agent at the starting point, and signing in his presence, a certificate to that effect, which, when signed by the Secretary at Rome, will entitle the member to return at the rate of one cent per mile.

The outlook for a large attendance is promising. Every arrangement is being made to entertain the Association in the best style.

Be in Rome April 18th.

